# Concomitant Sjögren’s disease as a biomarker for treatment effectiveness in rheumatoid arthritis - results from the Swiss clinical quality management cohort

**DOI:** 10.1186/s13075-024-03302-z

**Published:** 2024-03-14

**Authors:** Lisa Christ, Seraphina Kissling, Axel Finckh, Benjamin A. Fisher, Sabine Adler, Britta Maurer, Burkhard Möller, Florian Kollert

**Affiliations:** 1 Department of Rheumatology and Immunology, University Hospital and University of Bern, Bern, Switzerland; 2 Statistics and Data Management Group, Swiss Clinical Quality Management Foundation, Zurich, Switzerland; 3 Division of Rheumatology, University Hospitals of Geneva, Geneva, Switzerland; 4 Institute of Inflammation and Ageing, College of Medical and Dental Sciences, University of Birmingham, Birmingham, UK; 5 Birmingham Biomedical Research Centre, Department of Rheumatology, National Institute for Health Research (NIHR), University Hospitals Birmingham NHS Foundation Trust, Birmingham, UK; 6 Clinic of Rheumatology and Immunology, Medical University Hospital Aarau, Aarau, Aargau, Switzerland

**Keywords:** Rheumatoid arthritis, Sjögren’s disease, Treatment response

## Abstract

**Objective:**

To investigate the clinical phenotype and treatment response in patients with rheumatoid arthritis (RA) with and without concomitant Sjögren’s disease (SjD).

**Methods:**

In this observational cohort study, patients with RA from the Swiss Clinical Quality Management in Rheumatic Diseases registry were categorised according to the presence or absence of SjD. To assess treatment effectiveness, drug retention of tumor necrosis factor-α-inhibitors (TNFi) was compared to other mode of action (OMA) biologics and Janus kinase-inhibitors (JAKi) in RA patients with and without SjD. Adjusted hazard ratios (HR) for time to drug discontinuation were compared in crude and adjusted Cox proportional regression models for potential confounders.

**Results:**

We identified 5974 patients without and 337 patients with concomitant SjD. Patients with SjD were more likely to be female, to have a positive rheumatoid factor, higher disease activity scores, and erosive bone damage. For treatment response, a total of 6781 treatment courses were analysed. After one year, patients with concomitant SjD were less likely to reach DAS28 remission with all three treatment modalities. Patients with concomitant SjD had a higher hazard for stopping TNFi treatment (adjusted HR 1.3 [95% CI 1.07–1.6]; OMA HR 1.12 [0.91–1.37]; JAKi HR 0.97 [0.62–1.53]). When compared to TNFi, patients with concomitant SjD had a significantly lower hazard for stopping treatment with OMA (adjusted HR 0.62 [95% CI 0.46–0.84]) and JAKi (HR 0.52 [0.28–0.96]).

**Conclusion:**

RA patients with concomitant SjD reveal a severe RA phenotype, are less responsive to treatment, and more likely to fail TNFi.

**Supplementary Information:**

The online version contains supplementary material available at 10.1186/s13075-024-03302-z.

## Introduction

Rheumatoid arthritis (RA) is an immune mediated inflammatory disease with predominant manifestations in the joints and may be accompanied by Sjögren’s disease (SjD) [1–8]. This subset of patients with polyautoimmunity, i.e. SjD and RA, remains poorly understood, underdiagnosed and undertreated.

Research of the last decades enabled the approval of multiple targeted therapies in RA. However, the variety of treatment options, including tumor necrosis factor-α-inhibition (TNFi), Interleukin-6 receptor-inhibition (IL-6Ri), depletion of CD20 positive B cells and Janus kinase-inhibition (JAKi), contrasts with a lack of personalised medicine. No validated biomarkers to enable individualised treatment are included in the current EULAR guidelines [5]. Hence, with the exception of autoantibody positivity for B cell depletion, variation in disease pathophysiology and phenotype do not guide treatment decisions.

RA patients with concomitant SjD (overlap patients) define a distinct clinical subset and are characterised by an aggressive and erosive RA phenotype [2, 9, 10]. Brown et al. compared 85 overlap patients to 744 patients with RA and demonstrated that patients with concomitant SjD had more erosions irrespective of RA disease duration, age, or seropositivity [9]. Two single center cohorts found higher disease activity and more frequent lung involvement in overlap patients (*n* = 74 and 85, respectively) [2, 9]. The largest study so far was derived from an US registry (Corrona RA) and revealed that overlap patients (*n* = 7870) were more likely to be female, seropositive, have a longer RA duration, higher disease activity and more erosive disease [4, 10].

That treatment responses might differ with regard to the presence of SjD was demonstrated in trials of epratuzumab (anti-CD22) in systemic lupus erythematosus (SLE), which only showed efficacy in the subgroup of patients with both SLE and SjD [11]. One single center study compared treatment response in 126 overlap patients (RA/SjD) to 126 RA only patients (propensity score matched (PSM)) and found that overlap patients were less likely to reach remission [12]. Accordingly, a PSM analysis using the Corrona RA registry revealed less reduction in Clinical Disease Activity Index (CDAI) and RA-related patient-reported outcomes at one year in 283 overlap patients [10]. However, response to different treatment modalities was not assessed in these studies.

The open-label ROSE trial assessed treatment response to abatacept in 36 overlap patients and found response to both RA and SjD symptoms after one year [13]. In RA, the presence of SSA/Ro antibodies was associated with an inferior clinical response to both, infliximab and abatacept [14].

SjD is characterised by high levels of interferon (IFN) α and BAFF and both are further elevated by TNFi treatment which failed to show clinical efficacy in SjD [15]. Since patients with RA and SjD overlap might exhibit stronger type I IFN and B cell activation compared with RA alone, it can be hypothesised that non TNFi-therapies might be superior in these patients. However, studies assessing the relative efficacy of different treatment modalities in RA in the presence or absence of SjD are lacking. We hypothesise that the clinical phenotype and treatment response differs in patients with concomitant SjD compared to RA alone with an inferior response to TNFi in contrast to other targeted therapies.

To test this hypothesis, we performed the comparative treatment effectiveness in RA with and without concomitant SjD (CoRASS) study. We analysed data from the Swiss Clinical Quality Management in Rheumatic Diseases (SCQM) registry, which collects clinical as well as imaging data longitudinally from hospital and practice based rheumatologists in Switzerland [16].

## Methods

### Study design and participants

This is a retrospectively conducted observational cohort study based on prospectively collected data from the SCQM registry (01.01.2000–01.01.2021) [16]. The study was approved by the local ethics committee (file number 2020–02274). Diagnosis of RA and SjD was based on the judgement of the treating rheumatologist. Patients were classified as having SjD if they were ever diagnosed. All RA patients with written informed consent and available clinical data were included. Patients with sicca symptoms but without SjD diagnosis were excluded.

To define the clinical phenotype, descriptive comparison was performed between RA patients with SjD and RA patients without sicca symptoms at the time of SCQM inclusion.

To assess treatment response, all treatment courses (TC) from patients with RA aged 18 years and older and treated with TNFi, other mode of action (OMA: abatacept, IL-6Ri, rituximab) or JAKi were assessed. All eligible TC of patients with SjD were classified into the overlap group. Treatment start had to be under SCQM follow-up. Patients with SjD were compared to patients without and in a second step, we assessed treatment response between different treatment modalities within the overlap patients.

### Outcomes

Time on treatment (= retention time) was assessed as the primary outcome and response after one year of treatment as a secondary outcome.

Response was assessed by Disease Activity Score-28 (DAS28)- C-reactive protein (CRP), a score of < 2.6 being defined as remission [17].

### Statistics

*P*-values for the descriptive comparison are from Fisher test for nominal and Kruskal test for continuous variables. *P*-values for the comparison of median time on treatment are from a log-rank test.

For the primary outcome, we estimated hazard ratios (HR) for drug discontinuation between the comparisons of interest using Cox models with a cluster term by patient to take into account that patients could contribute multiple treatment courses. We defined time on treatment as duration between first dose to the earliest of either end of drug exposure (last dose plus washout period, listed in the online supplementary Table 1) or the start (first dose) of a new biologic or targeted synthetic disease-modifying anti-rheumatic drug (b/tsDMARD). We treated the start of a new b/tsDMARD as the end of the drug’s exposure since parallel treatment with several b/tsDMARDs is uncommon. If neither of these events had occurred until the last visit of a patient, the time on treatment was right-censored at the patient’s last visit.

Adjustment of Cox models was performed for age, gender, seropositivity, years since RA diagnosis, years since study start, concomitant DMARDs, number of prior biologic treatments, smoking and body-mass index (BMI). Models containing years since study start were stratified for study period (2000–2008, 2008–2013 and 2013–2021). We further included models with additional adjustment for DAS28-CRP (model 2) and Health Assessment Questionnaire (HAQ, model 3). As a sensitivity analysis, etanercept was compared to the other TNFi and IL-6Ri to abatacept and rituximab.

For the secondary outcome, we estimated the odds ratios (OR) to reach remission (defined as DAS28-CRP < 2.6) in the comparisons of interest using logistic regression models in patients followed-up until the time-point of interest (1 year after start of treatment ± 6 months). In case of multiple follow-up visits within the defined time window, the visit closest to 1 year was selected. We analysed the response to treatment in patients under treatment at the time point of interest (i.e. similar to a per protocol (PP) set-up), and in addition as a response tolerance remission (RTR) analysis, in which patients who discontinue treatment before the time-point of interest due to adverse event or ineffectiveness are imputed as non-responders. Definition of RTR analysis is displayed in the online supplementary data 1. Generalized estimating equations logistic regression models with an exchangeable correlation matrix were used, to account for multiple treatment courses per patient. Data analysis was performed with the R language and environment for statistical computing (version 4.1.1) [18].

### Role of the funding source

The funder had no role in study design, data collection, data analysis, data interpretation, or writing of the manuscript.

## Results

**Fig. 1:**
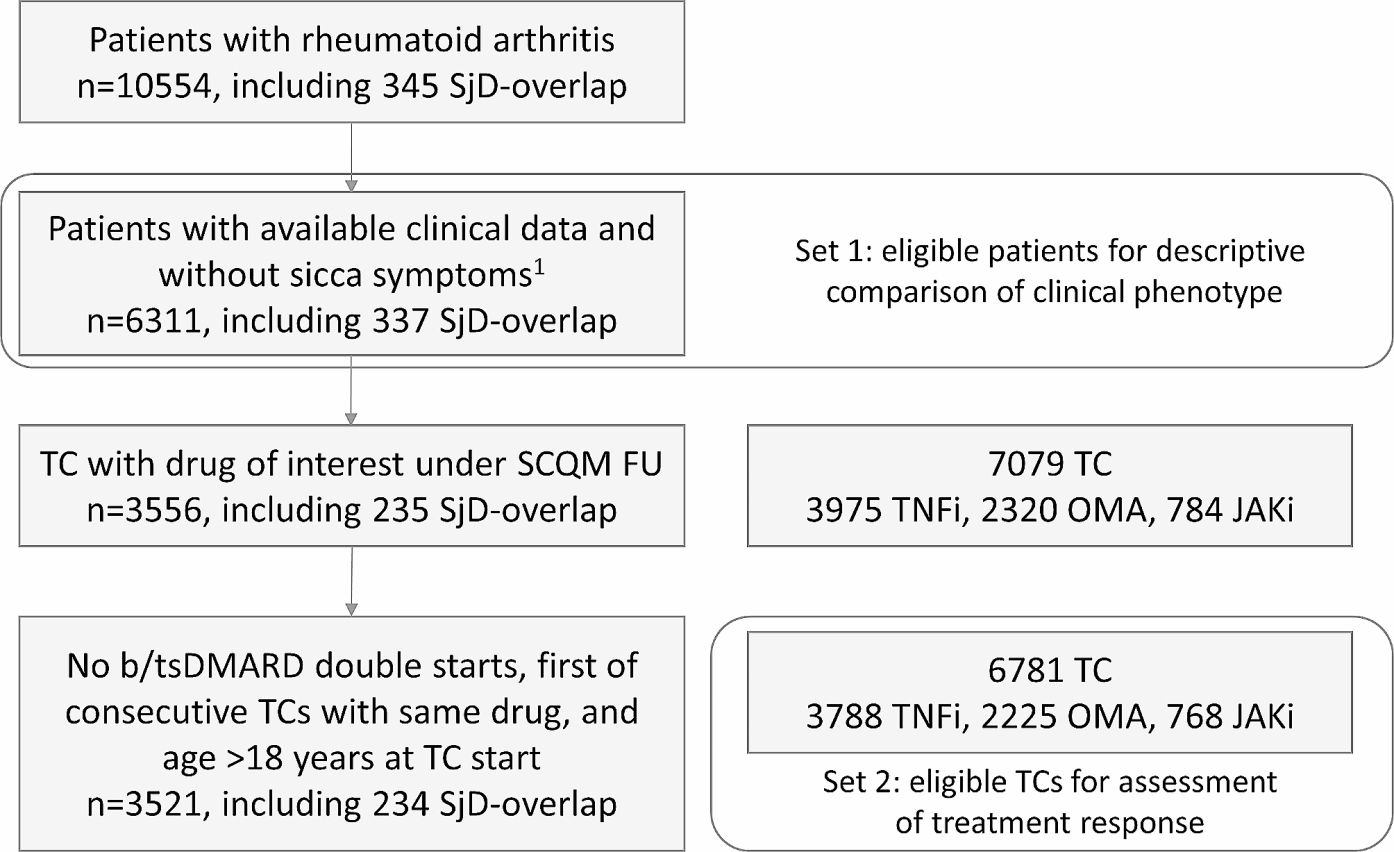
Flow chart of eligible patients and treatment courses after each inclusion step. ^1^Patients with sicca symptoms but without Sjögren’s disease diagnosis were excluded (*n* = 2417). b/tsDMARD, biologic/ targeted synthetic disease-modifying anti-rheumatic drug; FU, follow-up; JAKi, Janus kinase-inhibitor; OMA, other modes of action; SCQM, Swiss Clinical Quality Management in Rheumatic Diseases; SjD, Sjögren’s disease; TC, treatment course; TNFi, Tumour necrosis factor-inhibitor

For the descriptive comparison, we identified a total of 5974 RA without concomitant SjD and 337 overlap patients. Figure 1 displays the flow chart of eligible patients and TC after each inclusion step.

Patients with concomitant SjD were more likely to be female, non-smokers, to be rheumatoid factor (RF) positive, to have anti-cyclic citrullinated peptide antibodies, a longer RA disease duration, higher disease activity scores (DAS28, CDAI [5]), worse patient reported outcomes (HAQ or RA Disease Activity Index-5 (RADAI-5) score [19]), more signs of synovitis and a higher erosive burden (Ratingen score [20]) at the time of SCQM inclusion (Table 1). Ultrasound (Swiss Sonography in Arthritis and Rheumatism (SONAR) score [21]) revealed a higher power doppler (PD) but similar grey scale (GS) ultrasound scores in patients with overlap disease. Regarding prior treatment, these patients were more likely to have received previous glucocorticoid, rituximab, or abatacept treatment.

**Table 1:** Patient characteristics at inclusion

Variable	Levels	RA patients	RA/SjD patients	All patients	*p* value
**Number of patients**		5974	337	6311	
**Age** [years]		57 (47, 66)	56 (50, 64)	57 (47, 66)	0.76
**Gender**	Female	4227 (70.8)	298 (88.4)	4525 (71.1)	< 0.001
**Smoker** **(*n* = 4171)**	Current	1381 (34.9)	51 (23.4)	1432 (34.3)	
	Former	1088 (27.5)	59 (27.1)	1147 (27.5)	
	Never	1484 (27.5)	108 (49.5)	1592 (38.2)	< 0.001
**BMI** [kg/m^2^] (*n* = 5559)		25.1 (22.2, 28.7)	25.0 (22.5, 28.7)	25.1 (22.2, 28.7)	0.98
**Years since RA diagnosis** (*n* = 6190)		2.8 (0.7, 8.4)	4.9 (1.3, 13.2)	2.8 (0.7, 8.6)	< 0.001
**CCP** (*n* = 4605)	Positive	2836 (61.6)	201 (70.5)	3037 (62.2)	< 0.001
**RF** (*n* = 5772)	Positive	3826 (66.3)	252 (78.0)	4078 (66.9)	< 0.001
**Seropositivity**^a^ (*n* = 5889)		4175 (70.9)	271 (82.1)	4446 (71.5)	< 0.001
**CRP** [mg/l] (*n* = 3553)		5.0 (2.0, 10.5)	6.0 (2.0, 13.0)	5.0 (2.0, 11.0)	0.29
**ESR** [mm/h] (*n* = 5865)		16.0 (8.0, 30.0)	20.0 (10.0, 34.0)	16.0 (8.0, 30.0)	< 0.001
**Prior therapy**	Corticosteroids	1347 (22.6)	106 (31.4)	1453 (23.0)	< 0.001
	MTX (*n* = 6296)	3047 (51.0)	164 (48.7)	3211 (50.9)	0.20
	LEF (*n* = 6297)	1030 (17.2)	57 (16.9)	1087 (17.2)	0.01
	DMARD^b^	3492 (58.5)	200 (59.4)	3692 (58.5)	0.78
	TNFi (*n* = 6307)	1223 (20.5)	78 (23.1)	1301 (20.6)	0.09
	JAKi	118 (2.0)	5 (1.5)	123 (1.9)	0.69
	IL-6Ri	234 (3.9)	9 (2.7)	243 (3.9)	0.031
	Abatacept, RTX (*n* = 6285)	315 (5.3)	44 (13.1)	359 (5.7)	< 0.001
**Number of previous biologics**	0	4432 (74.2)	227 (67.4)	4659 (73.8)	
	1	1132 (18.9)	76 (22.6)	1208 (19.1)	
	2	253 (4.2)	22 (6.5)	275 (4.4)	
	≥ 3	157 (2.6)	12 (3.6)	169 (2.7)	0.03
**Current therapy**	Corticosteroids	2244 (37.6)	157 (46.6)	2401 (38.0)	0.001
	MTX	3267 (54.7)	175 (51.9)	3442 (54.5)	0.17
	LEF	882 (14.8)	37 (11.0)	919 (14.6)	0.003
	TNFi	1556 (26.1)	64 (19.0)	1620 (25.7)	0.003
	JAKi	147 (2.5)	2 (0.6)	149 (2.4)	0.20
	IL-6Ri	210 (3.5)	6 (1.8)	216 (3.4)	0.20
	Abatacept (*n* = 6284)	183 (61.2)	19 (43.2)	202 (58.9)	< 0.001
	RTX (*n* = 6284)	116 (38.8)	25 (56.8)	141 (41.1)	< 0.001
**HAQ** (*n* = 5432)		0.8 (0.2, 1.2)	1.0 (0.6, 1.5)	0.8 (0.2, 1.4)	< 0.001
**DAS28-CRP** (*n* = 3471)		3.3 (2.2, 4.3)	3.6 (2.4, 4.7)	3.3 (2.2, 4.3)	0.009
**DAS28-CRP remission**^c^ (*n* = 3307)		1055 (31.9)	44 (26.8)	1099 (31.7)	0.20
**DAS28-ESR** (*n* = 5781)		3.9 (2.7, 5.0)	4.4 (3.4, 5.4)	3.9 (2.7, 5.0)	< 0.001
**Patients global**^d^ (*n* = 5362)		4.0 (2.0, 6.0)	5.0 (3.0, 7.0)	4.0 (2.0, 6.0)	< 0.001
**Physicians global**^d^ (*n* = 5253)		3.0 (2.0, 5.0)	4.0 (2.9, 6.0)	3.0 (2.0, 5.0)	0.02
**Number of swollen joints**^e^ (*n* = 5754)		4.0 (1.0, 8.0)	5.0 (1.0, 10.0)	4.0 (1.0, 8.0)	< 0.001
**Number of tender joints**^e^ (*n* = 5742)		3.0 (0.0, 8.0)	6.0 (1.5, 11.0)	3.0 (0.0, 9.0)	< 0.001
**CDAI** (*n* = 4055)		16.0 (8.0, 27.0)	22.5 (14.0, 32.8)	17.0 (8.0, 28.0)	< 0.001
**SDAI** (*n* = 1709)		13.3 (6.2, 23.8)	16.2 (7.2, 26.7)	13.4 (6.2, 24.0)	0.25
**Ratingen score** (*n* = 4519)		10.0 (4.0, 20.0)	13.0 (6.0, 26.0)	10.0 (4.0, 20.0)	< 0.001
**Ultrasound score**	GS score (*n* = 607)	8.0 (4.0, 14.0)	11.0 (7.0, 14.2)	8.0 (5.0, 14.0)	0.08
	PD score (*n* = 561)	1.0 (0.0, 3.0)	3.0 (0.2, 6.0)	1.0 (0.0, 3.0)	0.003

Displayed are n (%) for nominal and median (Q1, Q3) for continuous variables (*n* = 6311 if not specified)

^a^Presence of either CCP or RF or both. ^b^Treatment with DMARD. This includes: azathioprine, hydroxychloroquine, cyclosporine, LEF, MTX, sulfasalazine. ^c^Remission defined as DAS28 < 2.6. ^d^Visual analog scale from 0 (best) to 10 (worst). ^e^28 joint count

BMI, body mass index; CCP, CCP-antibody; CDAI, clinical disease activity index; CRP, C-reactive protein; DAS28, disease activity score 28; DMARD, disease-modifying anti-rheumatic drug; ESR, erythrocyte sedimentation rate; GS, grey scale; HAQ, health assessment questionnaire; IL-6Ri, Interleukin-6-inhibitor; JAKi, Janus kinase-inhibitor; LEF, leflunomide; MTX, methotrexate; PD, power doppler; RA, rheumatoid arthritis; RF, rheumatoid factor; RTX, rituximab; SDAI, simplified disease activity index; SjD, Sjögren’s disease; TNFi, tumour necrosis factor-inhibitor

For the assessment of treatment effectiveness, we identified a total of 6781 (3788 TNFi, 2225 OMA, 768 JAKi) eligible TCs from 234 overlap patients and 3287 patients with RA alone. Out of the 6781 TC, 2967 TC were stopped, 1081 TC were stopped because another b/tsDMARD was started before the recorded stop date, and 2733 TC were censored at end of follow up. Stop reasons are documented in 2807 of the 2967 TC (no difference between RA and RA/SjD) and include: ineffectiveness (*n* = 2807), adverse events (*n* = 638), remission (*n* = 275), and other reasons, such as patient preference or pregnancy (*n* = 660). In 265/553 TC of RA/SjD patients, the SjD diagnosis was subsequent to the initiation of the respective TC. Baseline characteristics at TC-start are displayed in online supplementary Table 2.

**Fig. 2:**
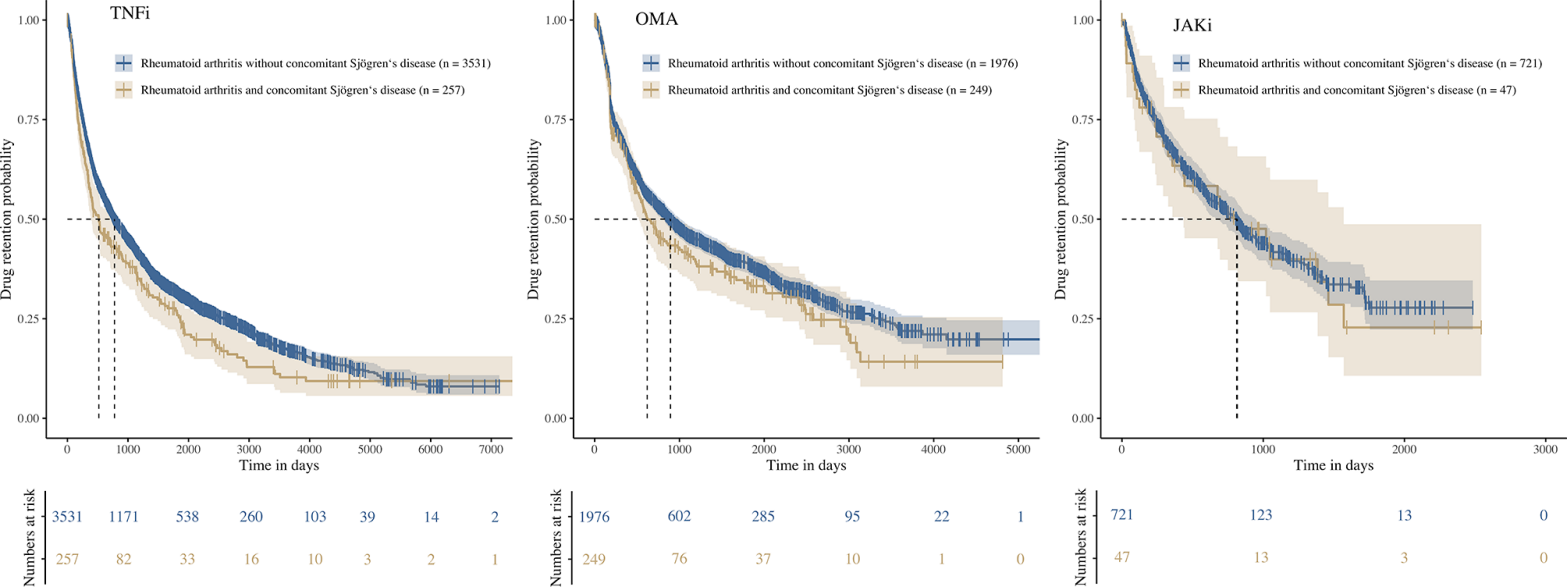
Kaplan-Meier plot of retention times for eligible TNFi, OMA, and JAKi treatment courses. JAKi, Janus kinase-inhibitor; OMA, other modes of action; TC, treatment course; TNFi, Tumour necrosis factor-inhibitor

Overall, RA/SjD patients displayed shorter drug retention rates as compared to RA patients without concomitant SjD (online supplementary Fig. 4). Median retention time for patients with overlap disease versus without SjD was 518 (95% confidence interval (CI) 391–721) versus 777 (728–866) days for TNFi, 619 (525–979) versus 894 (796–1016) days for OMA and 817 (436–1570) versus 812 (684–949) days for JAKi (Fig. 2). In the unadjusted Cox model (number of events = 4048), there was evidence that overlap patients had a higher hazard for stopping TNFi treatment than patients without SjD (crude HR 1.24 [95% CI 1.06–1.44]). A trend was found for OMA (crude HR 1.16 [0.96–1.39]) and no difference was found for JAKi (crude HR 1.06 [0.71–1.57]). After adjustment (number of events = 2917), the hazard for stopping TNFi treatment was still higher in the overlap group (HR 1.30 [95% CI 1.07–1.60]). No difference was observed for OMA (HR 1.12 [0.91–1.37]) and JAKi (HR 0.97 [0.62–1.53]). Within the overlap group (number of events = 382), there was no evidence for a higher hazard for stopping JAKi compared to TNFi (crude HR 0.88 [95% CI 0.58–1.31]) in the unadjusted Cox model (patient characteristics at TC-start and Kaplan-Meier plot of retention times are displayed in the online supplementary Fig. 1 and online supplementary Table 3). We found a trend for OMA compared to TNFi (crude HR 0.82 [0.66–1.01]), which was not significant. After adjustment (number of events = 260), the hazard for stopping treatment was lower for OMA (HR 0.62 [95% CI 0.46–0.84]) and JAKi (HR 0.52 [0.28–0.96]) when compared to TNFi.

**Fig. 3:**
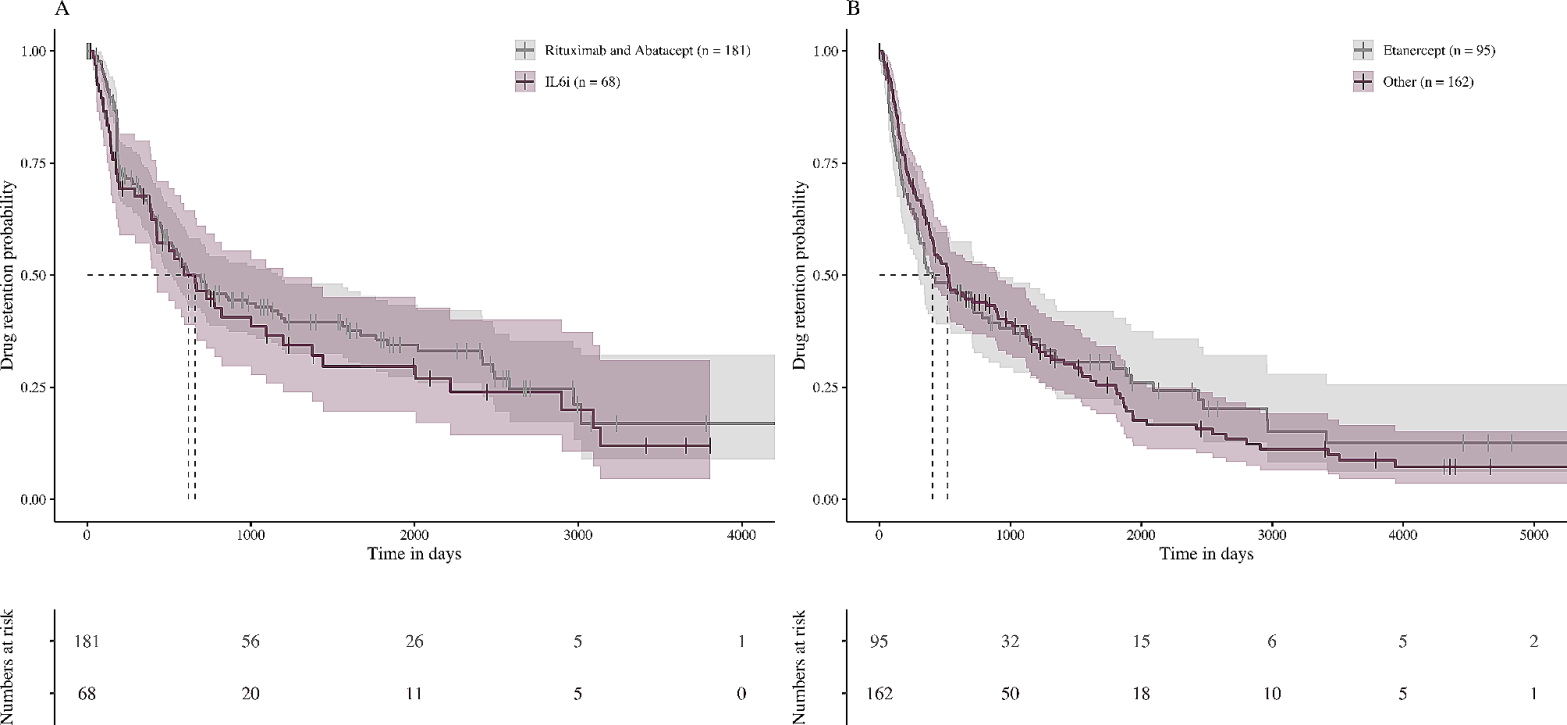
Kaplan-Meier plot of retention times for patients with rheumatoid arthritis and concomitant Sjögren’s disease. **A**, Eligible abatacept, rituximab, and IL-6Ri treatment courses. **B**, Eligible etanercept and other tumour necrosis factor-inhibitor treatment courses. IL-6i, Interleukin-6 receptor-inhibitor

The drug retention rates of overlap patients did not differ between etanercept as compared to the other TNFi and IL-6Ri as compared to rituximab and abatacept (Fig. 3). Median retention time was 405 (95% CI 301–919) days for etanercept and 521 (95% CI 410–902) days for the other TNFi (crude HR 1.036 [95% CI 0.76–1.41]) and 659 (95% CI 423–1197) days for IL-6Ri and 619 (95% CI 518–1166) days for rituximab and abatacept (crude HR 1.156 [0.81–1.65]), respectively. Drug retention of overlap patients differed between rituximab as compared to TNFi (online supplementary Fig. 5). We observed no differences in drug retention between overlap patients as compared to patients without SjD in model 2 including adjustment for DAS28-CRP levels (TNFi: 1.14 [95% CI 0.81–1.60], OMA: 1.10 [0.81–1.50], JAKi: 1.22 [0.67–2.22]) and in model 3 including adjustment for HAQ levels (TNFi: 1.24 [0.93–1.65], OMA: 0.97 [0.69–1.35], JAKi: 0.83 [0.46–1.49]).

For the secondary outcome, we assessed response at one year. The subset of eligible TCs included in the PP and RTR response analysis is displayed in the online supplementary Fig. 2.

**Fig. 4:**
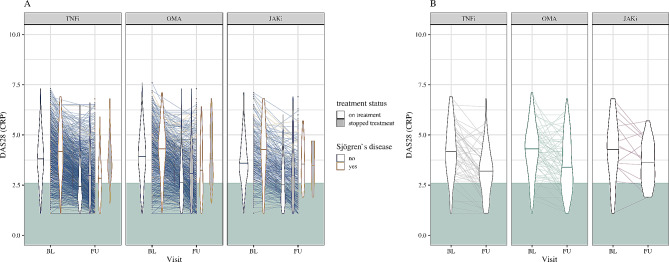
DAS28-CRP score at baseline and 1 year follow-up visit (response tolerance remission analysis). The distributions of DAS28-CRP values per group and timepoint are displayed as violin plots (density from lowest to highest value, median indicated by a horizontal line). **A**, Comparison of DAS28-CRP values between rheumatoid arthritis patients with and without concomitant Sjögren’s disease. **B**, Comparison of DAS28-CRP values between different treatment modalities within the overlap group. Grey: in remission (DAS28 < 2.6). BL, baseline; CRP, C-reactive protein; DAS28, Disease Activity Score-28; FU, follow-up; JAKi, Janus kinase-inhibitor; OMA, other modes of action; TNFi, Tumour necrosis factor-inhibitor

At one year, overlap patients receiving TNFi, OMA, or JAKi were less likely to reach DAS28 remission as compared to patients without concomitant SjD in the PP analysis (unadjusted model). After adjustment, overlap patients had inferior remission rates with JAKi and OMA, but not with TNFi treatment in the PP dataset (online supplementary Fig. 3 and online supplementary Table 4). The RTR analysis revealed that overlap patients were less likely than patients without associated SjD to reach DAS28 remission with TNFi (crude HR 2.12 [95% CI 1.35–3.34]), OMA (HR 1.71 [1.15–2.55]), or JAKi (HR 2.81 [1.16–6.83], Fig. 4). This was also true after adjustment (TNFi HR 2.0 [95% CI 1.22–3.28]; OMA HR 1.77 [1.17–2.7]; JAKi HR 3.49 [1.18–10.26]). Online supplementary Fig. 6 displays individual DAS28-CRP scores over time.

Within overlap patients, no difference was observed in reaching DAS28 remission for JAKi or OMA as compared to TNFi (PP and RTR analysis, unadjusted and adjusted model, Fig. 4, online supplementary Fig. 7 and online supplementary Table 5).

## Discussion

This study shows that patients with RA and concomitant SjD are more likely to fail TNFi treatment. Median drug retention of TNFi was substantially lower in patients with associated SjD (518 versus 777 days). If SjD was present, OMA treatment was associated with a 38% odds reduction and JAKi treatment with a 48% odds reduction to stop treatment as compared to TNFi. Given the fact that up to one third of patients with RA have concomitant SjD [4] and that B-cell or IFN-targeted treatment approaches might also be beneficial for treatment of SjD, non-TNFi treatment strategies may be considered early in patients with SjD overlap.

We chose drug retention as the primary outcome as follow-up data on remission outcomes (such as DAS28) had more missing values in our cohort and was hence more prone to bias. Due to the effect of SjD on the erythrocyte sedimentation rate (ESR), we chose DAS28-CRP to assess treatment response [17]. With regard to the remission outcome, RTR analysis provided more meaningful understanding compared to PP analysis in our study, as patients who discontinued the treatment prior to the timepoint of remission assessment were included as either non-responders (stop reason ineffectiveness or adverse event) or responders (stop reason remission). In the RTR analysis, we found that patients with concomitant SjD were less likely to reach DAS28 remission with all assessed treatment modalities. This is in line with the fact that RA/SjD patients have a more aggressive arthritis phenotype and a lower probability of reaching remission [10, 12].

SSA/Ro antibodies can be found in 3–17% of patients with RA and are found in the majority of SjD patients [22, 23]. Protein microarray analysis identified SSA autoantibodies as a predictive marker for the development of anti-drug antibodies (ADA) in patients with RA treated with adalimumab (64.3% vs. 4.3%, *p* < 000.1) [22]. The occurrence of ADA is less likely with etanercept as compared to the other TNFi [24]. The development of ADA is associated with inferior retention rates. To investigate whether the inferior retention rate of TNFi in our study might be related to the higher frequency of ADA in overlap patients, we compared drug retention rates of etanercept as compared to the other TNFi. However, there was no difference in retention rates (Fig. 3). Hence, it can be postulated that ADA might not be the primary driver for inferior TNFi retention in these patients. To assess whether poor treatment response to TNFi is responsible for the shorter drug retention, we assessed DAS28 remission outcome at one year. However, we did not observe less DAS28 remission with TNFi as compared to OMA or JAKi in our study. Due to missing data for DAS28 in our cohort, these results have to be interpreted with caution. Worsening of SjD might have contributed to shorter retention rates of TNFi. However, data on SjD outcomes and biomarkers of IFN or B-cell activity are not available in the SCQM registry and we found no difference in drug retention times for IL-6Ri as compared to B-cell or T-cell costimulation-targeted approaches (rituximab, abatacept).

The prevalence of SjD in our cohort (5.3%) was closer to the results reported by He et al. (14.5%) and Brown et al. (10%), but lower as compared to the US registry (30%) [2, 4, 9]. The US registry captured patients with clinical signs and symptoms of dry eyes, and/or dry mouth unrelated to medications, as having SjD [4]. RF was less common in the SjD subset of the US study (difference < 5% between both groups, in our study 12% difference) [4]. The lower prevalence of SjD in our cohort as compared to the US cohort might be explained by including only patients with a physician’s diagnosis of SjD rather than sicca symptoms alone. Notably, sicca symptoms are common in the general population and non-Sjögren’s causes are much more common. The advantage of our approach is that the applied diagnostic criteria are more specific, which may lead to a better quality of the cohort (percentage of correct diagnosis) but also to a potential underestimation of prevalence.

In our multicenter study population, patients with concomitant SjD demonstrated a female predominance, longer disease duration, more seropositivity, and revealed a more aggressive phenotype including higher disease activity, more synovitis and erosive disease in line with prior studies [2, 4, 9, 10]. We found that not only patient reported outcomes but also objective signs of synovitis in ultrasound contributed to the higher disease activity observed in this subset of patients.

Brown et al. demonstrated that overlap patients had a higher Sharp score, i.e. more erosive burden, which is in line with our findings [9]. Data on ultrasound in these patients is limited. One study found that patients with concomitant SjD were less likely to reach ultrasound remission (defined as GS ≤ 1 and PD = 0, max score 66 each) [25]. In our study, PD scores were higher and we observed a trend for a higher GS score (*p* = 0.08) in overlap patients.

The limitations of this study include the observational study design and the fact that we relied on the physician’s diagnosis for the definition and SjD might be underdiagnosed in patients with RA. This, however, is also an advantage, as it reflects the real-life clinical setting. To reduce the obstacle of a false negative SjD diagnosis, we excluded patients with reported sicca-symptoms but without a physician’s diagnosis of SjD. Strengths of our study include the large sample size, the detailed characterisation of our patients, and the multicenter, real-world study setting.

## Conclusions

CoRASS is the first study to assess the treatment response to different treatment modalities in RA with regard to the presence or absence of SjD. Patients with concomitant SjD revealed a more aggressive phenotype including higher disease activity, a higher ultrasound PD-score, and more erosive disease in this multicenter cohort. After one year, patients with concomitant SjD are less likely to reach DAS28 remission with all three treatment modes: TNFi, OMA, and JAKi. These patients are more likely to fail TNFi treatment and hence, patients with RA should be assessed for the presence of SjD and non-TNFi treatment modalities may be considered early.

### Electronic supplementary material

Below is the link to the electronic supplementary material.

Supplementary Material 1

## Data Availability

Research data and other materials will be available to the scientific community after publication. Restrictions apply to the availability of these data. Data was obtained from the Swiss Clinical Quality Management in Rheumatic Diseases (SCQM) and its availability requires approval and permission from the license holder (SCQM). All requests should be submitted to the corresponding author for consideration.

## Abbreviations

ADA: Anti-drug antibodies
BMI: Body-mass index
CDAI: Clinical Disease Activity Index
CoRASS: Coparative Treatment effectiveness in Rheumatoid Arthritis with and without Sjögren`s disease
CRP: C-reactive protein
DAS28: Disease Activity Score-28
DMARD: Disease-modifying anti-rheumatic drug
ERS: Erythrocyte sedimentation rate
EULAR: European Alliance of Associations for Rheumatology
GS: Grey scale
HAQ: Health Assessment Questionnaire
HR: Hazard rations
IL-6Ri: Interleukin-6 receptor inhibitor
JAKi: Janus kinase-inhibitors
OMA: Other mode of action
PD: Power doppler
PP: Per protocol
RA: Rheumatoid Arthritis
RF: Rheumatoid factor
RTR: Response tolerance remission
SCQM: Swiss Clinical Quality Management in Rheumatic Diseases registry
SjD: Sjögren’s disease
SLE: Systemic Lupus Erthematosis
SONAR: Swiss Sonography in Arthritis and Rheumatism
TC: Treatment course
TNFi: Tumor necrosis factor-α-inhibitor
