# Supplementary material for: Concomitant Sjögren’s disease as a biomarker for treatment effectiveness in rheumatoid arthritis - results from the Swiss clinical quality management cohort
Source: Arthritis Res Ther. 2024 Mar 14;26:68. doi: 10.1186/s13075-024-03302-z (PMC10938669; doi:10.1186/s13075-024-03302-z)
Supplement: Supplementary file 1 — Supplementary Material 1 [file 13075_2024_3302_MOESM1_ESM.docx]

**Supplementary appendix**

Supplement to: Concomitant Sjögren’s disease as a biomarker for treatment effectiveness in rheumatoid arthritis - results from the Swiss Clinical Quality Management cohort

Table of contents

[**Supplementary figures** 2](#_Toc133847128)

[Supplementary Figure 1. Kaplan Meier plot of retention times of patients with rheumatoid arthritis and concomitant Sjögren’s disease 2](#_Toc133847129)

[Supplementary Figure 2. Number of treatment courses available for each type of response analysis 3](#_Toc133847130)

[Supplementary Figure 3. DAS28-CRP score at baseline and 1 year follow-up visit (per protocol analysis) 4](#_Toc133847131)

[Supplementary Figure 4. Kaplan Meier plot of retention times for all eligible treatment courses 5](#_Toc133847132)

[Supplementary Figure 5. Kaplan Meier plot of retention times of overlap patients with rheumatoid arthritis and concomitant Sjögren’s disease 6](#_Toc133847133)

[Supplementary Figure 6. DAS28-CRP score over time 7](#_Toc133847134)

[Supplementary Figure 7. DAS28-CRP score at baseline and 1 year follow-up visit (within the Sjögren’s disease group, per protocol analysis) 8](#_Toc133847135)

[**Supplementary tables** 9](#_Toc133847136)

[Supplementary Table 1. Number of days the drug is still considered active after the last dose. 9](#_Toc133847137)

[Supplementary Table 2. Patient characteristics of patients with rheumatoid arthritis with and without concomitant Sjögren’s disease at the start of an eligible treatment course. 10](#_Toc133847138)

[Supplementary Table 3. Patient characteristics of patients with rheumatoid arthritis and concomitant Sjögren’s disease at the start of an eligible treatment course. 11](#_Toc133847139)

[Supplementary Table 4. Odds ratios between patients with rheumatoid arthritis with and without concomitant Sjögren’s disease for the odds of not reaching DAS28-remission after one year (per protocol analysis). 12](#_Toc133847140)

[Supplementary Table 5. Odds ratios between treatment modalities for the odds of not reaching DAS28-remission after one year. Data for patients with rheumatoid arthritis and concomitant Sjögren’s disease. 12](#_Toc133847141)

[Supplementary Data 1. Response tolerance remission 12](#_Toc133847142)

# Supplementary figures

# Supplementary Figure 1. Kaplan Meier plot of retention times of patients with rheumatoid arthritis and concomitant Sjögren’s disease


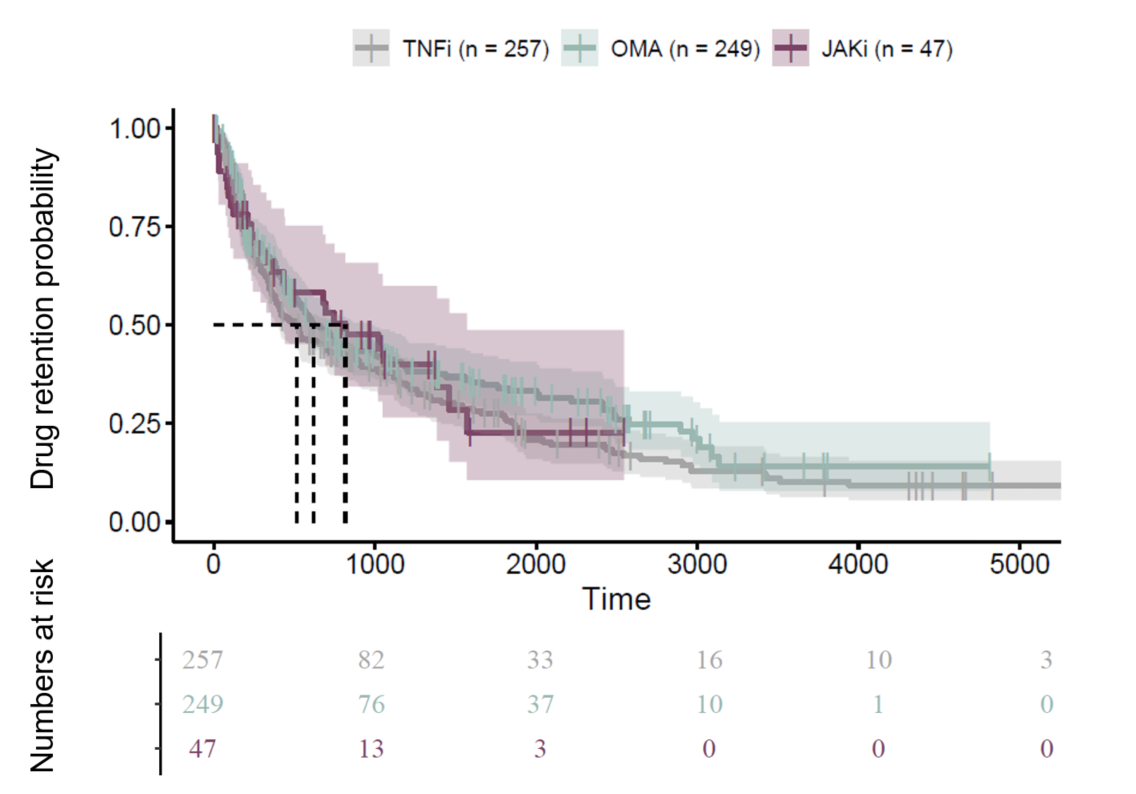


Kaplan Meier plot of retention times for eligible treatment courses of patients with RA and concomitant Sjögren’s disease (n=553). Patients characteristics at start of each treatment course are displayed in Supplementary table 3. Median retention time was 518 (TNFi, 95% CI 391-721), 619 (OMA, 95% CI 525-979), and 817 (JAKi, 95% CI 436-1570) days, respectively. The p-value corresponds to the log-rank test.

JAKi, Janus kinase-inhibitor; OMA, other modes of action; RA, rheumatoid arthritis. TNFi, Tumour necrosis factor-inhibitor.

Supplementary Figure 2. Number of treatment courses available for each type of response analysis


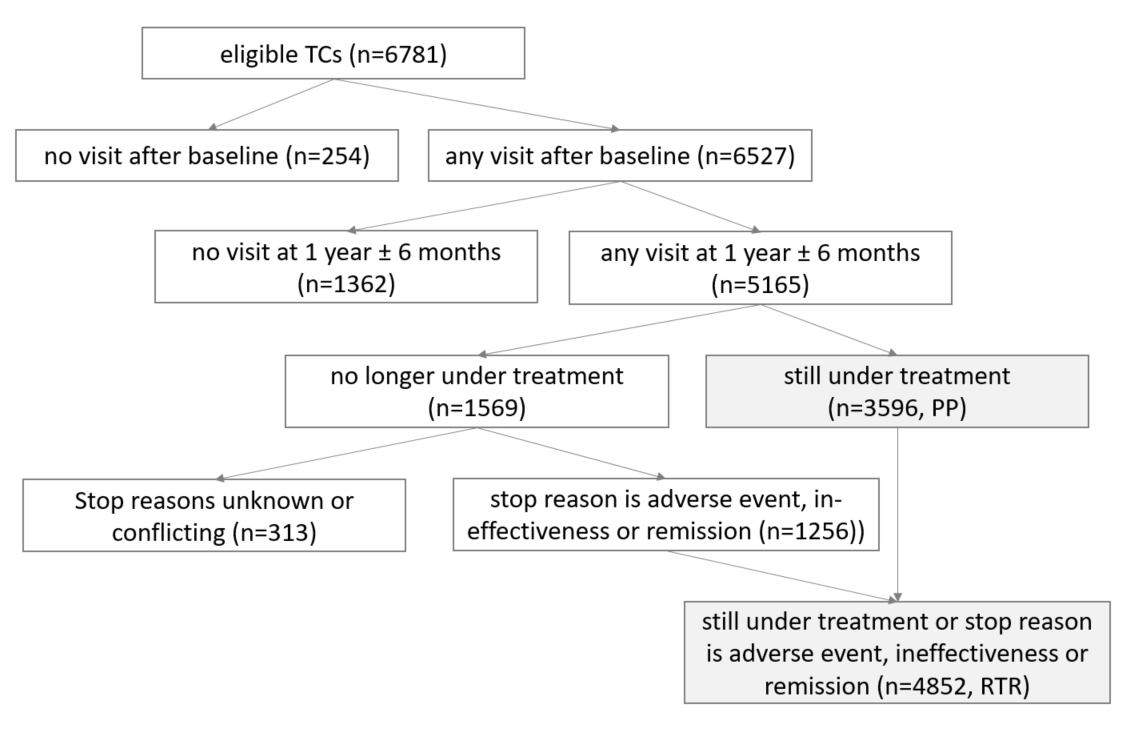


Out of the 4852 TC available for the response tolerance remission analysis, 3671 TC had available DAS28 values. For the per protocol analysis, this was true for 2415 out of 3596 TC.

DAS28, disease activity score 28; PP, per protocol; RTR, response tolerance remission; TC, treatment course.

# Supplementary Figure 3. DAS28-CRP score at baseline and 1 year follow-up visit (per protocol analysis)


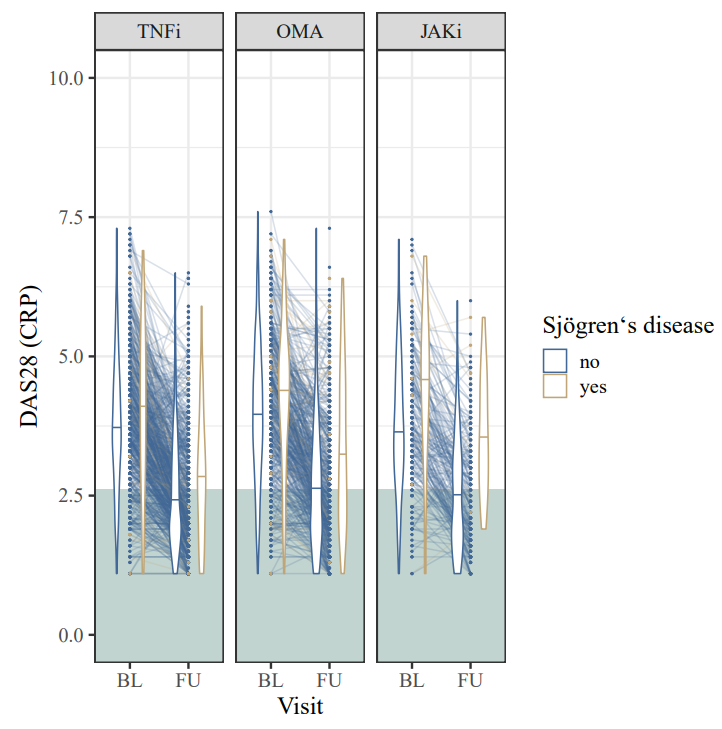
Comparison of DAS28-CRP values between rheumatoid arthritis patients with and without concomitant Sjögren’s disease (n=2415). The distributions of DAS28-CRP values per group and timepoint are displayed as violin plots (density from lowest to highest value, median indicated by a horizontal line). Green: in remission (DAS28 < 2.6).

BL, baseline; CRP, C-reactive protein; DAS28, Disease Activity Score-28; FU, follow-up; JAKi, Janus kinase-inhibitor; OMA, Other Modes of Action; TNFi, Tumour necrosis factor-inhibitor.

# Supplementary Figure 4. Kaplan Meier plot of retention times for all eligible treatment courses


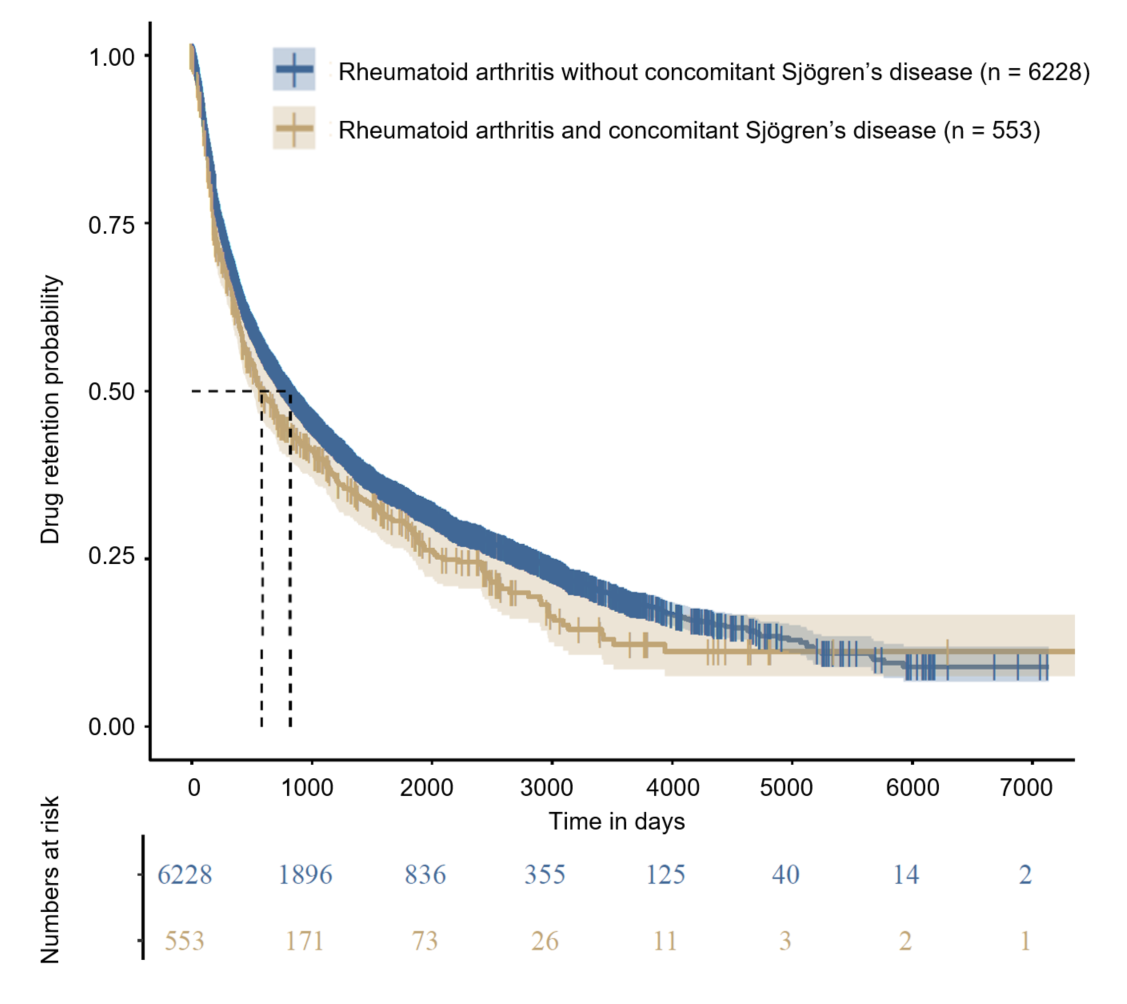


# Supplementary Figure 5. Kaplan Meier plot of retention times of overlap patients with rheumatoid arthritis and concomitant Sjögren’s disease


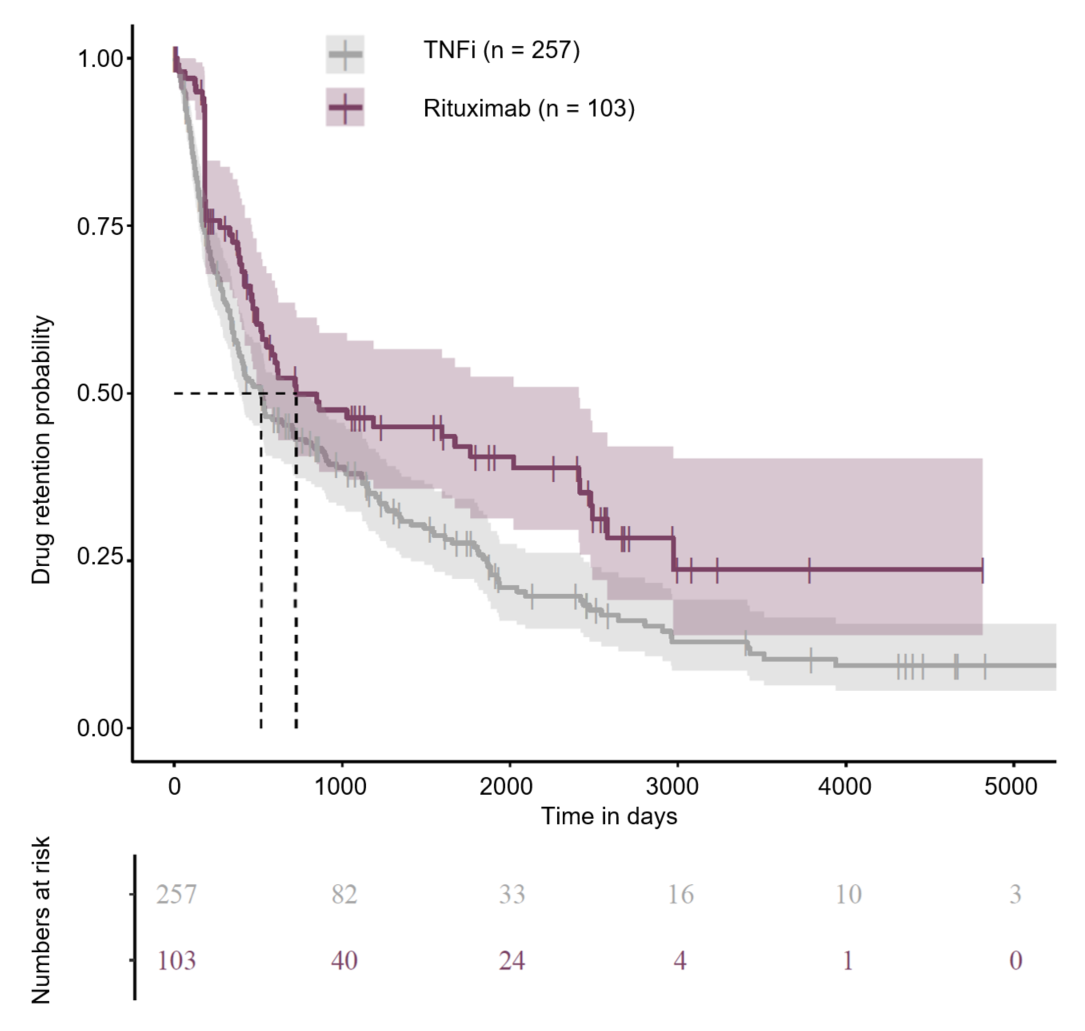


Median retention time is 728 (95% CI 525-2409) days for rituximab and 518 (95% CI 391-721) days for TNFi.

TNFi, Tumour necrosis factor-inhibitor.

# Supplementary Figure 6. DAS28-CRP score over time


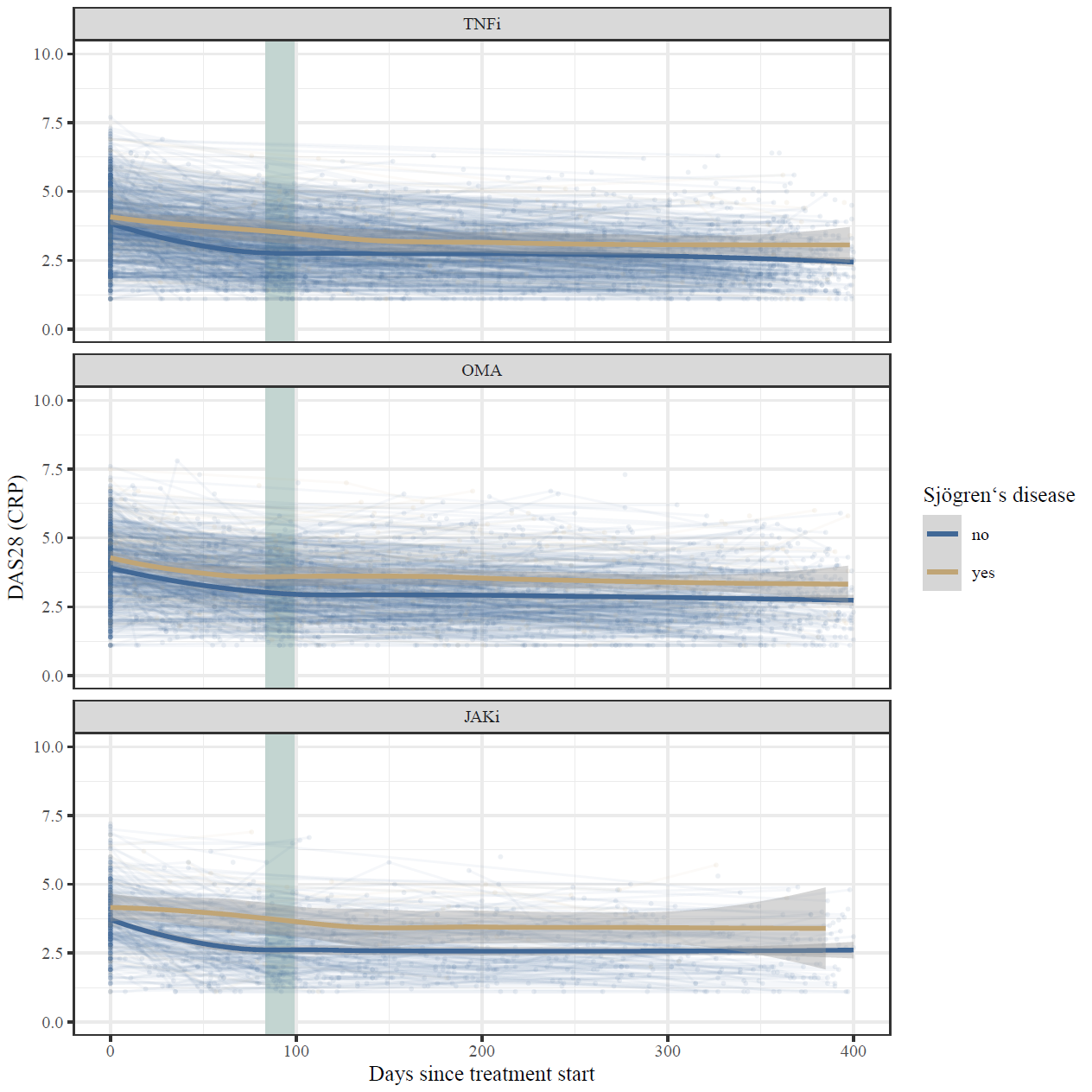


Display of individual DAS28-CRP values between rheumatoid arthritis patients with and without concomitant Sjögren’s disease over time. The bold line (blue/yellow) represents the loess regression line. Green bar: 3 months follow-up visit.

CRP, C-reactive protein; DAS28, Disease Activity Score-28; JAKi, Janus kinase-inhibitor; OMA, Other Modes of Action; TNFi, Tumour necrosis factor-inhibitor.

# Supplementary Figure 7. DAS28-CRP score at baseline and 1 year follow-up visit (within the Sjögren’s disease group, per protocol analysis)


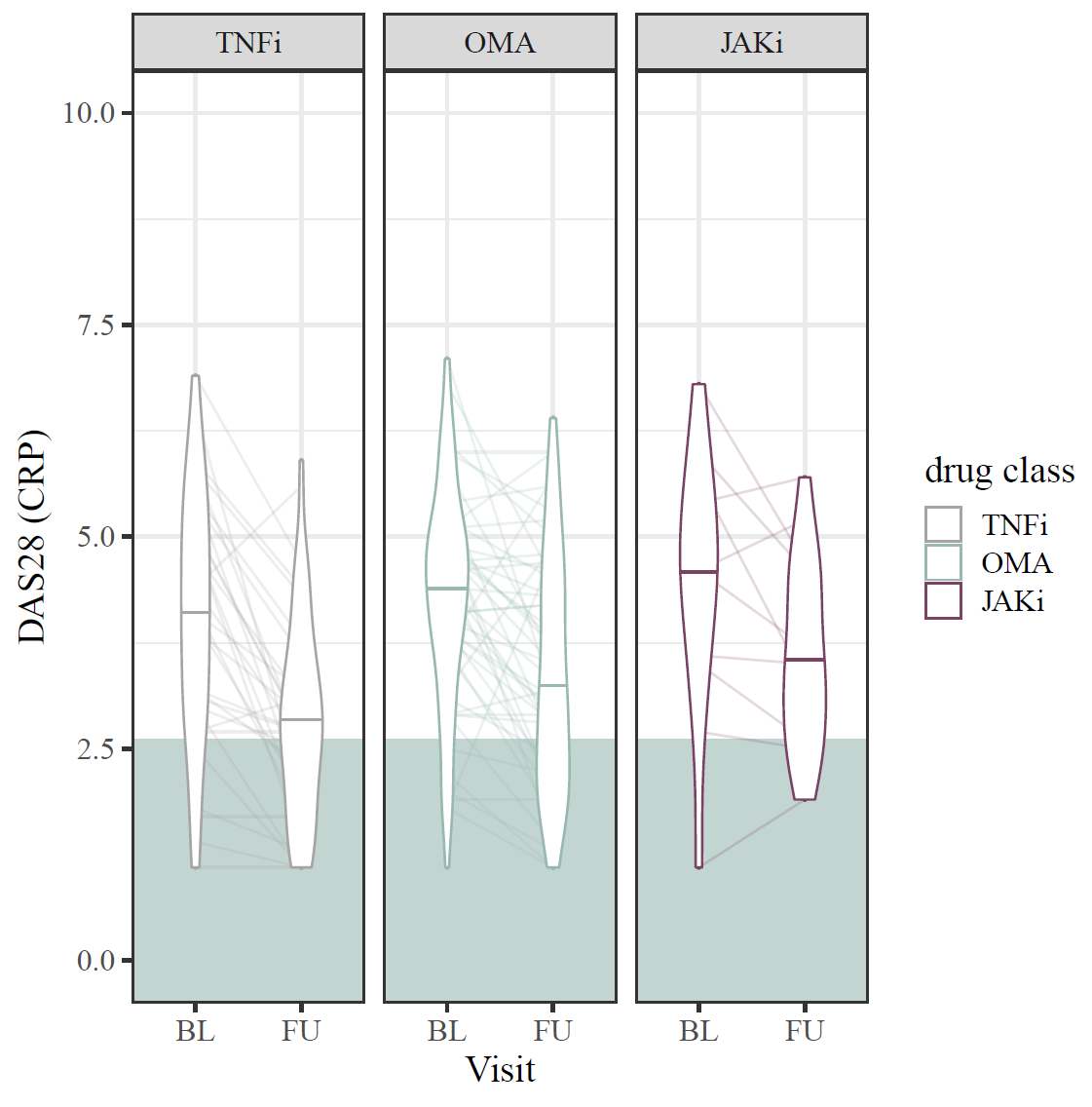
Comparison of DAS28-CRP values between different treatment modalities within the Sjögren’s disease group (n=186). The distributions of DAS28-CRP values per group and timepoint are displayed as violin plots (density from lowest to highest value, median indicated by a horizontal line). Green: in remission (DAS28 < 2.6).

BL, baseline; CRP, C-reactive protein; DAS28, Disease Activity Score-28; FU, follow-up; JAKi, Janus kinase-inhibitor; OMA, Other Modes of Action; TNFi, Tumour necrosis factor-inhibitor.

# Supplementary tables

# Supplementary Table 1. Number of days the drug is still considered active after the last dose.

| **Drug** | **Route** | **Drug type** | **Washout period** |
| --- | --- | --- | --- |
| Abatacept | s.c. | OMA | 7 days |
| Abatacept | i.v. | OMA | 28 days |
| Adalimumab | s.c. | TNFi | 14 days |
| Barictinib | oral | JAKi | 1 day |
| Certolizumab | s.c. | TNFi | 14 days |
| Etanercept | s.c. | TNFi | 7 days |
| Golimumab | s.c. | TNFi | 28 days |
| Infliximab | i.v. | TNFi | 56 days (8 weeks) |
| Rituximab | i.v. | OMA | 168 days (24 weeks) |
| Sarilumab | s.c. | OMA | 14 days |
| Tocilizumab | i.v. | OMA | 28 days |
| Tocilizumab | s.c. | OMA | 7 days |
| Tofacitinib | oral | JAKi | 1 day |
| Upadacitinib | oral | JAKi | 1 day |

i.v., intravenous; JAKi, Janus kinase-inhibitior; OMA, Other mode of action; s.c., subcutaneous; TNFi, Tumour necrosis factor-inhibitior.

# Supplementary Table 2. Patient characteristics of patients with rheumatoid arthritis with and without concomitant Sjögren’s disease at the start of an eligible treatment course.

| **Variable** | **Levels** | **RA patients** | **RA/SjD patients** | **All patients** | **p value** |
| --- | --- | --- | --- | --- | --- |
| **Number of TC** |  | 6228 | 553 | 6781 |  |
| **Age** [years] |  | 56·6 (46·7-65·0) | 60·6 (52·1-67·4) | 56·9 (47·0-65·3) | <0·01 |
| **Gender** | Female | 4649 (74·7) | 481 (87·0) | 5130 (75·7) | <0·01 |
| **Smoker** (n=5266) | Current | 1319 (27·2) | 63 (15·0) | 1382 (26·2) |  |
|  | Former | 1471 (30·4) | 129 (30·6) | 1600 (30·4) |  |
|  | Never | 2055 (42·4) | 229 (54·4) | 2284 (43·4) | <0·01 |
| **BMI** [kg/m^2^] (n=6168) |  | 25·2 (22·1-29·0) | 25·3 (22·9-28·7) | 25·2 (22·2-29·0) | 0·42 |
| **Years since RA diagnosis** (n=6676) | | 6·5 (2·6-13·1) | 10·7 (4·4-20·1) | 6·8 (2·7-13·6) | <0·01 |
| **Years since study start** | | 12·8 (8·8·-16·3) | 11·1 (7·6-14·4) | 12·7 (8·6-16·2) | <0·01 |
| **CCP** (n=5776) | Positive | 3547 (66·9) | 351 (73·7) | 3898 (67·5) | <0·01 |
| **Rheumatoid factor** (n=6622) | Positive | 4307 (70·8) | 444 (82·2) | 4751 (71·8) | <0·01 |
| **Seropositivity**^a^ (n=6727) |  | 4658 (75·4) | 469 (85·6) | 5127 (76·2) | <0·01 |
| **Drug class** | TNFi | 3531 (56·7) | 257 (46·5) | 3788 (55·9) |  |
|  | JAKi | 721 (11·6) | 47 (8·5) | 768 (11·3) |  |
|  | OMA | 1976 (31·7) | 249 (45·0) | 2225 (32·8) | <0·01 |
| **Concomitant DMARD therapy** (n=6779) | None | 2046 (32·9) | 221 (40·0) | 2267 (33·4) |  |
|  | Other than MTX^b^ | 1402 (22·5) | 107 (19·4) | 1509 (22·3) |  |
|  | Including MTX^c^ | 2278 (44·6) | 225 (40·7) | 3003 (44·3) | <0·01 |
| **Concomitant corticosteroid therapy** (n=6778) | Yes | 2331 (37·5) | 301 (54·4) | 2632 (38·8) | <0·01 |
| **Number of previous biologics** (n=6778) | 0 | 2624 (42·1) | 176 (31·8) | 2800 (41·3) |  |
|  | 1 | 1736 (27·9) | 151 (27·3) | 1887 (27·8) |  |
|  | 2 | 974 (15·7) | 97 (17·5) | 1071 (15·8) |  |
|  | ≥3 | 871 (14·3) | 129 (23·3) | 1020 (15·1) | <0·01 |
| **HAQ** (n=3295) |  | 0·9 (0·4-1·5) | 1·4 (0·9-1·9) | 1·0 (0·4-1·5) | <0·01 |
| **DAS28-CRP** (n=2869) |  | 3·8 (3·0-4·7) | 4·3 (3·2-5·1) | 3·8 (3·0-4·7) | <0·01 |
| **Ratingen score** (n=176) |  | 15·0 (7·0, 38·5) | 16·0 (7·0, 37·0) | 15·5 (7·0, 38·2) | 0·99 |
| **Ultrasound score** | GS score (n=306) | 14·0 (8·0, 22·0) | 11·5 (8·2, 19·0) | 13·0 (8·0, 21·8) | 0·90 |
|  | PD score (n=281) | 2·0 (0·0, 6·0) | 6·0 (1·5, 13·0) | 3·0 (0·0, 7·0) | 0·02 |

Patient characteristics at the start of an eligible treatment course. Displayed are n (%) for nominal and median (Q1-Q3) for continuous variables (n=6781 if not specified). ^a^Presence of either CCP or rheumatoid factor or both. ^b^Treatment with DMARD other than MTX at start of TC. This includes: azathioprine, hydroxychloroquine, cyclosporine, leflunomide, sulfasalazine. ^c^Treatment with DMARD including MTX at start of TC.

BMI, body-mass index; CCP, CCP-antibody; CRP, C-reactive protein; DAS28, disease activity score 28; DMARD, disease-modifying antirheumatic drug; GS score, grey scale score; HAQ, health assessment questionnaire; JAKi, Janus kinase-inhibitor; MTX, methotrexate; OMA, other modes of action; PD score, power doppler score; RA, rheumatoid arthritis; SjD, Sjögren’s disease; TC, treatment course; TNFi, Tumour necrosis factor-inhibitor.

# Supplementary Table 3. Patient characteristics of patients with rheumatoid arthritis and concomitant Sjögren’s disease at the start of an eligible treatment course.

| **Variable** | **Levels** | **TNFi** | **OMA** | **JAKi** | **All patients** | **p value** |
| --- | --- | --- | --- | --- | --- | --- |
| **Number of TC** |  | 257 | 249 | 47 | 553 |  |
| **Age** [years] |  | 58·4 (49·7-64·7) | 62·2 (53·8-68·5) | 67·2 (55·6-72·5) | 60·6 (52·1-67·4) | <0·01 |
| **Gender** | Female | 225 (87·5) | 216 (86·8) | 40 (85·1) | 481 (87·0) | 0·84 |
| **Smoker** (n=421) | Current | 27 (16·0) | 32 (15·3) | 4 (9·3) | 63 (15·0) |  |
|  | Former | 45 (26·6) | 70 (33·5) | 14 (32·6) | 129 (30·6) |  |
|  | Never | 97 (57·4) | 107 (51·2) | 25 (58·1) | 229 (54·4) | 0·50 |
| **BMI** [kg/m^2^] (n=505) |  | 24·7 (21·9-28·4) | 25·7 (23·2-28·7) | 25·6 (22·9-30·9) | 25·3 (22·9-28·7) | 0·12 |
| **Years since RA diagnosis** (n=541) | | 8·7 (3·4-18·9) | 10·9 (4·8-19·9) | 13·9 (8·3-25·5) | 10·7 (4·4-20·1) | <0·01 |
| **Years since study start** | | 7·9 (5·3-12·2) | 12·3 (9·5-15·2) | 17·1 (14·5-18·1) | 11·1 (7·6-14·4) | <0·01 |
| **CCP** (n=476) | Positive | 153 (73·2) | 164 (73·5) | 34 (77·3) | 351 (73·7) | 0·89 |
| **Rheumatoid factor** (n=540) | Positive | 205 (82·3) | 200 (81·6) | 39 (84·8) | 444 (82·8) | 0·91 |
| **Seropositivity**^a^ (n=548) |  | 219 (85·9) | 209 (85·0) | 41 (87·2) | 469 (85·6) | 0·93 |
| **Calendar period** | 2000-05 | 58 (22·6) | 1 (0·4) | 0 | 59 (10·7) |  |
|  | 2005-10 | 110 (42·8) | 70 (28·1) | 0 | 180 (32·5) |  |
|  | 2010-15 | 65 (25·3) | 113 (45·4) | 14 (29·8) | 192 (34·7) |  |
|  | 2015-21 | 24 (9·3) | 65 (26·1) | 33 (70·2) | 122 (22·1) | <0·01 |
| **Concomitant DMARD therapy** | None | 90 (35·0) | 104 (41·8) | 27 (57·5) | 221 (40·0) |  |
|  | Other than MTX^b^ | 38 (14·8) | 59 (23·7) | 10 (21·3) | 107 (19·4) |  |
|  | Including MTX^c^ | 129 (50·2) | 86 (34·5) | 10 (21·3) | 225 (40·7) | <0·01 |
| **Concomitant corticosteroid therapy** | Yes | 136 (52·9) | 139 (55·8) | 26 (55·3) | 301 (54·4) | 0·80 |
| **Number of previous biologics** | 0 | 134 (52·1) | 37 (14·9) | 5 (10·6) | 176 (31·8) |  |
|  | 1 | 67 (26·1) | 80 (32·1) | 4 (8·5) | 151 (27·3) |  |
|  | 2 | 26 (10·1) | 67 (26·9) | 4 (8·5) | 97 (17·5) |  |
|  | ≥3 | 30 (11·7) | 65 (26·1) | 34 (72·3) | 129 (23·3) | <0·01 |
| **HAQ** (n=233) |  | 1·2 (0·8-1·9) | 1·4 (0·9-1·8) | 1·5 (0·9-1·8) | 1·4 (0·9-1·9) | 0·88 |
| **DAS28-CRP** (n=194) |  | 4·1 (3·2-5·1) | 4·3 (3·4-5·1) | 4·4 (3·3-5·1) | 4·3 (3·2-5·1) | 0·79 |
| **Ratingen score** (n=25) |  | 16·0 (7·5-39·5) | 9·0 (4·5-13·5) | NA | 16·0 (7·0-37·0) | 0·34 |
| **Ultrasound score** | GS score (n=30) | 20·0 (15·0-29·0) | 9·5 (6·5-11·8) | 13·0 (9·0-23·5) | 11·5 (8·2-19·0) | 0·03 |
|  | PD score (n=27) | 7·0 (5·0-14·2) | 2·0 (1·0-6·2) | 12·0 (5·5-21·5) | 6·0 (1·5-13·0) | 0·07 |

Displayed are n (%) for nominal and median (Q1-Q3) for continuous variables (n=553 if not specified). ^a^Presence of either CCP or rheumatoid factor or both. ^b^Treatment with DMARD other than MTX at start of TC. This includes: azathioprine, hydroxychloroquine, cyclosporine, leflunomide, sulfasalazine. ^c^Treatment with DMARD including MTX at start of TC.

BMI, body-mass index; CCP, cyclic citrullinated peptide-antibody; CRP, C-reactive protein; DAS28, disease activity score 28; DMARD, disease-modifying antirheumatic drug; GS score, grey scale score; HAQ, health assessment questionnaire; JAKi, Janus kinase-inhibitor; MTX, methotrexate; OMA, other modes of action; PD score, power doppler score; RA, rheumatoid arthritis; TC, treatment course; TNFi, Tumour necrosis factor-inhibitor.

# Supplementary Table 4. Odds ratios between patients with rheumatoid arthritis with and without concomitant Sjögren’s disease for the odds of not reaching DAS28-remission after one year (per protocol analysis).

|  | **Crude odds ratio** | 95% CI |
| --- | --- | --- |
| TNF-inhibitor | 1·94 | 1·13-3·4 |
| Other mode of action | 1·92 | 1·23-3·03 |
| JAK-inhibitor | 6·04 | 1·68-33·05 |
|  | **Adjusted odds ratio** | 95% CI |
| TNF-inhibitor | 1·58 | 0·89-2·81 |
| Other mode of action | 1·67 | 1·05-2·65 |
| JAK-inhibitor | 10·59 | 1·67-67·01 |

Reference: Rheumatoid arthritis without concomitant Sjögren’s disease.

DAS28, disease activity score 28; JAK, Janus kinase; TNF, Tumour necrosis factor.

# Supplementary Table 5. Odds ratios between treatment modalities for the odds of not reaching DAS28-remission after one year. Data for patients with rheumatoid arthritis and concomitant Sjögren’s disease.

| **Per protocol analysis** | | |
| --- | --- | --- |
|  | **Crude odds ratio** | 95% CI |
| Other mode of action | 1·24 | 0·62-2·49 |
| JAK-inhibitor | 3·37 | 0·84-19·9 |
|  | **Adjusted odds ratio** | 95% CI |
| Other mode of action | 0·96 | 0·33-2·83 |
| JAK-inhibitor | 5·98 | 0·60-59·75 |
| **Response tolerance remission analysis** | | |
|  | **Crude odds ratio** | 95% CI |
| Other mode of action | 0·75 | 0·4-1·38 |
| JAK-inhibitor | 1·43 | 0·43-6·22 |
|  | **Adjusted odds ratio** | 95% CI |
| Other mode of action | 0·51 | 0·19-1·38 |
| JAK-inhibitor | 1·32 | 0·09-19·28 |

Reference: Tumour necrosis factor-inhibitor.

DAS28, disease activity score 28; JAK, Janus kinase.

# Supplementary Data 1. Response tolerance remission

Analysis of people still on the same treatment and of people not anymore on the initial treatment who stopped treatment due to adverse event, ineffectiveness or remission. People who stopped treatment before due to adverse event or ineffectiveness are treated as non-responders and people who stopped due to remission are treated as responders.
